# The Role of Attitudes Toward Medication and Treatment Adherence in the Clinical Response to LAIs: Findings From the STAR Network Depot Study

**DOI:** 10.3389/fpsyt.2021.784366

**Published:** 2021-12-16

**Authors:** Andrea Aguglia, Laura Fusar-Poli, Andrea Amerio, Valeria Placenti, Carmen Concerto, Giovanni Martinotti, Giuseppe Carrà, Francesco Bartoli, Armando D'Agostino, Gianluca Serafini, Mario Amore, Eugenio Aguglia, Giovanni Ostuzzi, Corrado Barbui, The STAR Network Investigators

**Affiliations:** ^1^Department of Neuroscience, Rehabilitation, Ophthalmology, Genetics, Maternal and Child Health, Section of Psychiatry, Istituto di Ricovero e Cura a Carattere Scientifico (IRCCS) Ospedale Policlinico San Martino, University of Genoa, Genoa, Italy; ^2^Istituto di Ricovero e Cura a Carattere Scientifico (IRCCS) Ospedale Policlinico San Martino, Genoa, Italy; ^3^Department of Clinical and Experimental Medicine, Psychiatry Unit, University of Catania, Catania, Italy; ^4^Department of Neuroscience, Imaging and Clinical Sciences, “G. d'Annunzio” University of Chieti, Chieti, Italy; ^5^Department of Medicine and Surgery, University of Milano-Bicocca, Monza, Italy; ^6^Division of Psychiatry, University College London, London, United Kingdom; ^7^Department of Health Sciences, Ospedale San Paolo, University of Milan, Milan, Italy; ^8^World Health Organization (WHO) Collaborating Centre for Research and Training in Mental Health and Service Evaluation, Department of Neuroscience, Biomedicine and Movement Sciences, Section of Psychiatry, University of Verona, Verona, Italy

**Keywords:** long-acting injectable, antipsychotics, drug, attitude, adherence, therapeutic alliance, psychotic symptoms

## Abstract

**Background:** Long-acting injectable (LAI) antipsychotics are efficacious in managing psychotic symptoms in people affected by severe mental disorders, such as schizophrenia and bipolar disorder. The present study aimed to investigate whether attitude toward treatment and treatment adherence represent predictors of symptoms changes over time.

**Methods:** The STAR Network “Depot Study” was a naturalistic, multicenter, observational, prospective study that enrolled people initiating a LAI without restrictions on diagnosis, clinical severity or setting. Participants from 32 Italian centers were assessed at three time points: baseline, 6-month, and 12-month follow-up. Psychopathological symptoms, attitude toward medication and treatment adherence were measured using the Brief Psychiatric Rating Scale (BPRS), the Drug Attitude Inventory (DAI-10) and the Kemp's 7-point scale, respectively. Linear mixed-effects models were used to evaluate whether attitude toward medication and treatment adherence independently predicted symptoms changes over time. Analyses were conducted on the overall sample and then stratified according to the baseline severity (BPRS < 41 or BPRS ≥ 41).

**Results:** We included 461 participants of which 276 were males. The majority of participants had received a primary diagnosis of a schizophrenia spectrum disorder (71.80%) and initiated a treatment with a second-generation LAI (69.63%). BPRS, DAI-10, and Kemp's scale scores improved over time. Six linear regressions—conducted considering the outcome and predictors at baseline, 6-month, and 12-month follow-up independently—showed that both DAI-10 and Kemp's scale negatively associated with BPRS scores at the three considered time points. Linear mixed-effects models conducted on the overall sample did not show any significant association between attitude toward medication or treatment adherence and changes in psychiatric symptoms over time. However, after stratification according to baseline severity, we found that both DAI-10 and Kemp's scale negatively predicted changes in BPRS scores at 12-month follow-up regardless of baseline severity. The association at 6-month follow-up was confirmed only in the group with moderate or severe symptoms at baseline.

**Conclusion:** Our findings corroborate the importance of improving the quality of relationship between clinicians and patients. Shared decision making and thorough discussions about benefits and side effects may improve the outcome in patients with severe mental disorders.

## Introduction

Severe mental illnesses are at higher risk of relapses associated with increased re-hospitalization rates, suicide attempts, behavioral abnormalities, persistent positive and residual symptoms, higher cognitive impairment, partial response with longer time to achieve remission, worsen quality of life and social functioning, and greater mortality and morbidity (1–5). A partial/absence adherence to pharmacological treatment is considered one of the most relevant risk factors for relapses (6), and it is influenced by several patient-, illness-, medication-, clinician-, and environmental-related factors (7–13).

It is well-known that oral and long-acting injectable (LAI) antipsychotics are similarly tolerable (14) and effective in the treatment of severe mental illnesses. Nevertheless, there is a literature debate on this topic, with randomized trials in clinically stable individuals showing a similar efficacy profile (15), and with large observational studies favoring LAI over oral antipsychotics in terms of re-hospitalizations (16–18). Observational studies might be less restrictive in terms of patient selection and have higher external validity, although an increased risk of detection, performance and prescribing bias may represent a relevant limitation (19). Although head-to-head comparison of LAIs did not show relevant differences between LAI treatments, aripiprazole, olanzapine, and paliperidone (1- and 3-monthly formulation) might be considered first-line choices, as they are supported by the highest certainty of evidence for both relapse prevention and acceptability (20). Furthermore, benefits of LAI over oral formulation include improved bioavailability, maintenance stable plasma levels and receptor occupancy avoiding daily peaks, reducing plasma fluctuation, and increasing safety and tolerability, higher relapse prevention, greater compliance and earlier detection of non-adherence, requirement of regular visits with a psychiatrist specialist (21–23).

Given this evidence, guidelines did not provide any recommendations on which LAIs should be preferred but underlined the importance to reconsider these formulations for acute and long-term maintenance treatment rather than later stages of illness (24–26), even if the European rate of LAIs use in daily clinical practice is generally lower than 30–40% (27–32). Several barriers are recognized by clinicians, summarized as follows: needs of trained medical practitioners, poor *in vitro-in vivo* correlation, high cost, longer time to achieve maximum concentration, possibility of dose dumping, fear of injection, and perception of LAIs as painful and intrusive, difficult retrieval of drug in case of toxicity, increased variability among dosage unit, conceptualization that LAIs are only for chronic patients, perception of LAIs as punitive and coercive, subjective fear of not being able to control the dosage, self-stigma related to severe mental illness diagnosis and antipsychotics prescription (22, 32–35).

Recently, LAIs have been largely destigmatized by clinicians, and several strategies to increase LAIs use have been suggested (35, 36). They include drug-related interventions, such as simplified regimens and prevention of potential side effects to ameliorate adherence, patient-related interventions (i.e., psychosocial, behavioral, and personalized interventions), and service-related interventions (i.e., emerging technology-based monitoring, mobile applications, and artificial intelligence-based communications). In this context, a significant role is given by the so-called “shared decision-making,” with a more patient-inclusive approach, determining a balanced reasoned decision with a negotiation between clinician and patient (37, 38).

Leveraging data from a large observational, longitudinal, multicenter study involving individuals starting any LAI medication, the present study aimed to investigate whether attitudes toward treatment and adherence to therapy predicted changes in psychiatric symptomatology over time.

## Materials and Methods

### Sample

This study was conducted according to STROBE (STrengthening the Reporting of OBservational studies in Epidemiology) statement items (39). The STROBE statement-checklist is provided in the Supplementary Table 1.

This is a large pragmatic, multicenter, observational, and longitudinal study, involving individuals starting any LAI medication. The detailed description of materials and methods of STAR Network Depot Study is provided on the first published paper (40). Another recently published paper considered follow-up data from the baseline recruitment phase (41).

The inclusion criteria for participants were: age ≥ 18 years, starting a LAI medication, not taking any other LAI during the previous 3 months and willing to sign the written informed consent for voluntary participation. In particular, patients were consecutively recruited over a period of 12 months and assessed at 6- and 12-month follow-up. Individuals from any clinical setting, with any psychiatric diagnosis and any concomitant pharmacological treatment, were included in this study to trace the daily clinical activity in real-world. Participating centers are in- and out- patient services, including hospital psychiatric wards, mental health, and community day centers, residential facilities, part of the Italian STAR Network (*Servizi Territoriali Associati per la Ricerca -* Community Services Associated for Research), aiming to provide original data from real-world clinical practice (42, 43).

All participants signed a written informed consent prior their recruitment into the study, to have their clinical data potentially used for teaching and research purposes (provided that these data are anonymously treated). The study design was conducted in accordance with the guidelines provided in the current version of the Declaration of Helsinki (44). The protocol was approved by the local Ethics Committees of all participating centers and is publicly available at the Open Science Framework (OSF) online repository (https://osf.io/wt8kx/). The STAR Network Depot Study was not supported by any industry funding neither any monetary remuneration was provided for included subjects.

### Assessment

A semi-structured interview used in previous published studies was administered to collect the socio-demographic characteristics, clinical, and pharmacological information (40, 41, 45, 46). Clinical interviews, health records and chart reviews were used to collect participants' baseline data while a follow-up form was used to collect psychopathological and pharmacological data.

In addition, specific psychometric tools were administered to investigate clinical dimensions, attitude toward medications and overall adherence to treatment, as follows: first, the clinician-rated Brief Psychiatric Rating Scale (BPRS) is validated in the Italian language by Roncone et al. (47) and assesses the overall level of psychiatric symptoms with the following severity: mild (31–40 total score), moderate (41–52 total score) and severe (above 52 total score) (48). This evaluation scale reported a high inter-rater reliability in both raters with high and low clinical experience (49). Second, the self-administered Drug Attitude Inventory 10 items (DAI-10) is validated in the Italian language (50) and assesses the patient attitude toward medications with a total score ranging from −10 to 10. A higher total score indicates a better overall attitude toward medications with a positive attitude defined as a DAI-10 total score > 0. Lastly, the clinician-rated Kemp's 7-point scale (51) is administered by the clinician and assesses overall adherence to treatment with a total score ranging from 1 to 7. A higher total score indicates a better overall adherence to treatment with good acceptance defined as Kemp's total score ≥ 5.

All clinicians participating in the recruitment and evaluation of patients performed a training meeting on the procedures and rating scales used in the study. These psychometric tools were administered at baseline and after 6 and 12 months of follow-up.

### Statistical Analysis

Baseline and follow-up data were periodically forwarded from each recruiting center to the coordinating center (University of Verona), where they were entered into a computer database. Data correctness and consistency were ensured with the use of a double-entry technique and by applying a set of electronic and manual edit checks.

Continuous variables were expressed as means and standard deviations, whereas categorical variables were expressed as absolute numbers and percentages.

First, we conducted an ANOVA to evaluate the changes in the considered outcome (BPRS) and predictors (DAI-10, Kemp's 7-point scale) scales over time. Second, six linear regression models - including the BPRS as outcome and the DAI-10 and the Kemp's scale as predictors - were computed independently using scores at baseline, 6-month, and 12-month follow-up (i.e., each association between the predictors and the outcome was calculated at the same timepoint). All regression models were adjusted for age and sex. Finally, linear mixed-effects models (LMEs) were used to analyze whether attitude toward medication (DAI-10) and adherence to treatment (Kemp's 7-point scale) were associated with changes in BPRS scores over time. LMEs were adjusted for age and sex (fixed effects), center and participant (random effects). LMEs were conducted on the overall sample and then stratified according to baseline severity. Specifically, the sample was divided into two groups (baseline BPRS <41 or ≥41) based on the cut-offs proposed by Leucht et al. (48). The first group included patients with mild symptoms at baseline; the second group included patients with moderate or severe symptoms at baseline.

To reduce attrition bias, supplementary analyses using last observation carried forward (LOCF) approach were conducted by imputing the last observed scores on the collected variables (BPRS, DAI-10, and Kemp's scale). LOCF analyses were reported in Supplementary Tables 2, 3.

All statistical analyses and figures were carried out using Stata for Windows, version 16 (StataCorp, College Station, Texas, USA).

## Results

### Characteristics of Participants at Baseline

At baseline, the sample comprised 461 participants recruited in 32 Italian centers, of which 276 were males. Mean age was 41.72 ± 12.86, with ages ranging between 18 and 76 years. Participants were mostly unemployed (49.24%) and had never been married (70.28%). The majority of participants received a primary diagnosis of a schizophrenia spectrum disorder (71.80%) and initiated a treatment with a second-generation LAI (69.63%). Socio-demographic and clinical characteristics of the sample are reported in Table 1.

**Table 1:** Characteristics of the sample (*N* = 461).

Age, mean (SD)	41.72 (12.86)
Sex, male, *N* (%)	276 (59.87)
**Education, *N* (%)**	
Illiterate/no title	7 (1.52)
Primary school	28 (6.07)
Secondary school	194 (42.08)
Diploma	181 (39.26)
University	45 (9.76)
Unknown	6 (1.30)
**Marital status, *N* (%)**	
Never married	324 (70.28)
Married	67 (14.53)
Widowed	4 (0.87)
Separated/divorced	65 (14.09)
Unknown	1 (0.22)
**Employment, *N* (%)**	
Employed	102 (22.13)
Unemployed	227 (49.24)
Housewife	24 (5.21)
Student	16 (3.47)
Retired	68 (14.75)
Other (e.g., sheltered employment)	24 (5.21)
**Primary diagnosis, *N* (%)**	
Schizophrenia spectrum disorders	331 (71.80)
Mood disorders	84 (18.22)
Others	46 (9.98)
**Type of LAIs, *N* (%)**	
First-generation LAI antipsychotics	140 (30.37)
Second-generation LAI antipsychotics	321 (69.63)
**Concomitant oral medication, *N* (%)**	
None	38 (8.24)
1	99 (21.47)
2	156 (33.84)
3 or more	168 (36.44)
Substance abuse, *N* (%)	91 (19.74)
Alcohol abuse, *N* (%)	66 (14.32)

*LAI, Long-acting injectable*.

### Changes of Psychiatric Symptoms (BPRS), Attitude Toward Drugs (DAI), and Adherence to Treatment (Kemp's 7-Point Scale) Over Time

Multiple comparison ANOVA showed that psychiatric symptoms measured through the BPRS significantly improved over time [*F*_(2, 686)_ = 259.98, *p* < 0.001]. Analogously, DAI-10 scores significantly increased, indicating a more confident attitude of participants toward medication [*F*_(2, 678)_ = 22.78, *p* < 0.001]. Finally, Kemp's scale significantly increased at 6- and 12-month follow-up, showing a patients' better adherence to pharmacological treatment [*F*_(2, 686)_ = 20.02, *p* < 0.001]. Results are presented in Table 2.

**Table 2:** Repeated measures ANOVAs evaluating changes of the brief psychiatric rating scale (BPRS), drug attitude inventory (DAI-10), and Kemp's 7-point scale scores over time.

	**Baseline**		**6 months**		**12 months**			
	***N***	**Mean (SD)**	***N***	**Mean (SD)**	***N***	**Mean (SD)**	***F***	***p***
BPRS	461	48.96 (14.67)	357	36.50 (11.12)	331	35.48 (11.06)	259.98	<0.001
DAI-10	459	2.00 (5.33)	355	3.37 (5.05)	326	3.92 (5.07)	22.78	<0.001
Kemp's scale	460	4.79 (1.44)	357	5.25 (1.48)	332	5.33 (1.56)	20.02	<0.001

*BPRS, Brief Psychiatric Rating Scale; DAI-10, Drug Attitude Inventory; SD, Standard deviation*.

### Association Between Attitudes Toward Medication, Treatment Adherence, and Symptoms Improvement at Baseline, 6- and 12-Month Follow-Up

Linear regression analyses, adjusted for age and sex, showed a negative association between DAI-10 scores and BPRS scores at baseline [*B* = −0.57 (95% CI −0.82, −0.32)], 6-month [*B* = −0.63 (95% CI −0.85, −0.40)], and 12-month follow-up [*B* = −0.65 (95% CI −0.88, −0.43)], indicating that a more positive attitude toward medication was associated with less severe psychiatric symptoms at each time point independently. Additionally, the Kemp's 7-point scale was negatively associated with BPRS scores at baseline [*B* = −3.12 (95% CI −4.01, −2.22)], after 6 months [*B* = −2.87 (95% CI −3.63, −2.11)] and after 12 months [*B* = −2.97 (95% CI −3.66, −2.28)] of treatment with LAIs. These results indicate that a higher compliance to therapy was related to less severe symptoms at each timepoint separately.

**Figure 1:**
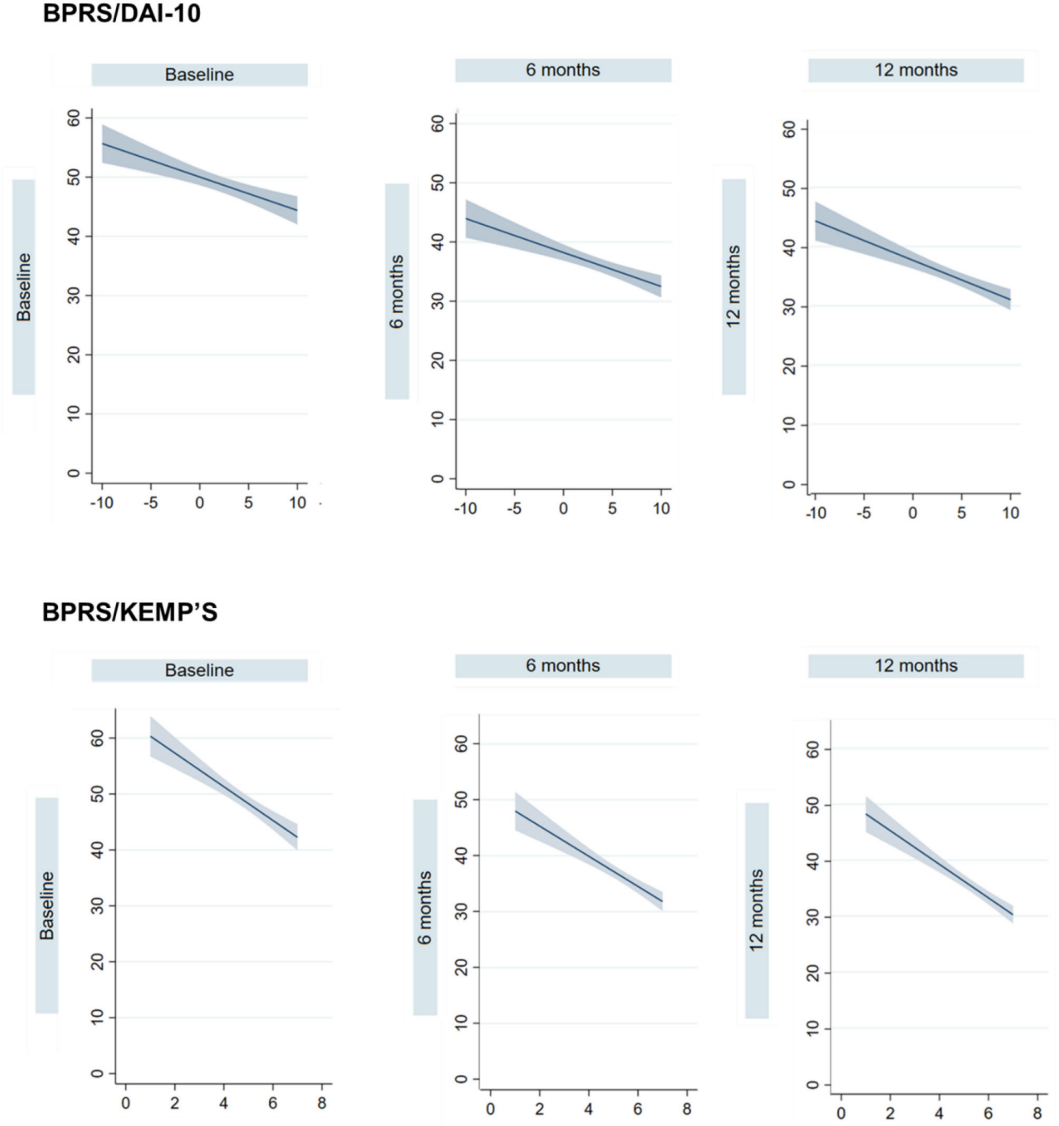
Unadjusted linear prediction lines and 95% confidence intervals (CI) of the Brief Psychiatric Rating Scale on the Drug Attitude Inventory-10 items (DAI-10) and the Kemp's 7-point scale at baseline, 6-month, and 12-month follow-up.

Figure 1 shows unadjusted linear prediction lines of BPRS on DAI-10 and Kemp's 7-point scale at baseline, 6-month, and 12-month follow-up.

### Association Between Attitudes Toward Medication, Treatment Adherence, and Change in Psychiatric Symptoms Over Time

Considering the overall sample, LMEs adjusted for age and sex showed no significant effects between the DAI-10 and the Kemp's 7-point scale and changes in BPRS scores (Table 3). These results were confirmed by the LOCF analyses (Supplementary Table 2).

**Table 3:** Linear mixed effect models to estimate whether attitude toward treatment and treatment adherence predict symptoms changes over time.

**BPRS**	**Linear mixed effect models**		
	***B***	**95% CI**	***P***
DAI-10 × (6 months vs. baseline)	−0.03	−0.29, 0.23	0.81
DAI-10 × (12 months vs. baseline)	−0.17	−0.44, 0.10	0.23
Kemp's × (6 months vs. baseline)	0.48	−0.43, 1.39	0.30
Kemp's × (12 months vs. baseline)	0.35	−0.57, 1.26	0.46

*Each model includes: BPRS as dependent variable, fixed effects: predictor (DAI-10 or Kemp's scale), time, predictor × time, age, and sex: random-effects: center, participant*.

*BPRS, Brief Psychiatric Rating Scale; DAI-10, Drug Attitude Inventory; Kemp's, Kemp's 7-point scale*.

After stratifying the sample according to baseline severity, DAI-10 was significantly associated with changes in BPRS scores at 6-month follow-up in the group with baseline BPRS ≥ 41 [*B* = −0.32 (95% CI −0.61, −0.03)] and at 12-month follow-up in both groups [BPRS < 41: *B* = −0.35 (95% CI −0.66, −0.05); BPRS ≥ 41: *B* = −0.41 (95% CI −0.71, −0.11)]. Similarly, Kemp's 7-point scale was associated with changes in BPRS scores at 6-month evaluation in the group with the greatest severity at baseline [*B* = −1.48 (95% CI −2.53, −0.43)], and after 12 months in both groups [BPRS < 41: *B* = −1.82 (95% CI −2.88, −0.77); BPRS ≥ 41: *B* = −1.46 (95% CI −2.51, −0.41)] (Table 4).

**Table 4:** Linear mixed effect models to estimate whether attitude toward treatment and treatment adherence predict symptoms changes over time, with sample stratified according to baseline severity (BPRS < 41 or BPRS ≥ 41).

	***B***	**95% CI**	***P***
**Baseline BPRS < 41 (*n* = 144)**			
DAI-10 × (6 months vs. baseline)	−0.05	−0.35, 0.24	0.74
DAI-10 × (12 months vs. baseline)	−0.35	−0.66, −0.05	0.02
Kemp's × (6 months vs. baseline)	−0.48	−1.51, 0.55	0.36
Kemp's × (12 months vs. baseline)	−1.82	−2.88, −0.77	0.001
**Baseline BPRS ≥ 41 (*n* = 317)**			
DAI-10 × (6 months vs. baseline)	−0.32	−0.61, −0.03	0.03
DAI-10 × (12 months vs. baseline)	−0.41	−0.71, −0.11	0.007
Kemp's × (6 months vs. baseline)	−1.48	−2.53, −0.43	0.006
Kemp's × (12 months vs. baseline)	−1.46	−2.51, −0.41	0.006

*Each model includes: BPRS as dependent variable, fixed effects: predictor (DAI-10 or Kemp's scale), time, predictor × time, age, and sex: random-effects: center, participant*.

*BPRS, Brief Psychiatric Rating Scale; DAI-10, Drug Attitude Inventory; Kem's, Kemp's 7-point scale*.

LOCF analyses confirmed only the findings related to the group including patients with moderate and severe symptoms at baseline. Contrarywise, the group with the mildest symptoms did not show any significant association between DAI-10 or Kemp's and changes in BPRS score over time in LOCF analyses (Supplementary Table 3).

## Discussion

The present study investigated the relationship between attitude toward medications, treatment adherence, and changes in psychiatric symptoms in a large sample of ordinary practice patients that received treatment with LAIs over a period of 1 year.

First, our study showed improvement of attitude toward medication and treatment adherence over the course of the study, alongside with improvement of psychiatric symptoms. This is in line with recent meta-analyses, showing benefits of LAIs prescription in early psychosis (52), as well as in the treatment maintenance of schizophrenia (18, 20), not only in research settings but also in real-world clinical practice. Second, our findings support an association between the considered predictors and severity of psychiatric symptoms at the three selected timepoints, when considered independently. Nevertheless, examining this prediction longitudinally in the overall sample, we found that neither attitude toward medication nor treatment adherence was associated with changes in psychiatric symptomatology at 6- and 12-month follow-up.

The characteristics of the recruited sample may represent a reason of this non-significant finding. A large majority of the patients included in our study were affected by schizophrenia spectrum disorders, a group of severe chronic conditions frequently associated with poor insight levels (53). Previous studies have reported that insight represents one of the most important factors influencing attitude toward treatment, with higher levels of insight associated to a more positive attitude (54, 55). Poor illness insight has also been related to low adherence to treatment in psychotic patients, according to a recent systematic review (13). Another potential explanation is related to the combined use of LAIs and oral medication in this study. In fact, a large majority of participants (91.8%) were taking concomitant oral psychotropic medications. If one hand LAIs are administered at regular intervals in outpatient services or at home under the supervision of a nurse and a psychiatrist, on the other hand the current compliance to oral medications was more difficult to control. Therefore, there might have been a discrepancy between the compliance to LAIs and to oral medications. Moreover, we cannot exclude that patient taking specific types of oral medication, such as supplementary antipsychotics or anticholinergics to alleviate extrapyramidal symptoms, may have had different responses on the individual attitude toward treatment or adherence to therapy. Indeed, side effects represent one of the most important reasons for LAI discontinuation in clinical practice (56). This notion has been confirmed also in this sample, as reported in our previous paper (41): reasons for discontinuation were mainly ascribed to the onset of adverse events (32.9%), followed by participant refusal of the medication (20.6%). Interestingly, discontinuers showed significantly lower DAI-10 and Kemp's 7-point scale than non-discontinuers.

From a methodological point of view, it is worth mentioning that while both the BPRS and the Kemp's 7-point scale are clinician-rated tool, the DAI-10 was completed by participants. Self-report tools might not be reliable in people affected by severe mental illnesses, particularly in psychotic patients (57). Thus, it is possible that attitude toward treatment was not adequately reported by patients included in our analysis. Moreover, more frequent follow-up visits (i.e., monthly) might have been useful to detect subtler changes, especially at the beginning of treatment with LAIs, when it is crucial to establish an optimal therapeutically alliance with patients who are initiating a new treatment. Nevertheless, given the naturalistic and pragmatic design of the study, the frequency of follow-up visits was widely variable across recruiting centers and could not be considered in our analysis.

It is interesting to note that, after stratifying the sample according to baseline severity, our results have changed. In fact, we found that improvement in both treatment adherence and attitude toward medication was associated with a significant reduction in BPRS scores after 12 months of treatment with LAI regardless of baseline severity. A significant negative association was also found after 6 months in the group with the greatest symptom severity at baseline. It is possible to hypothesize that the stratified analyses have minimized, at least in part, some of the inter-individual differences discussed above. Moreover, significant results appear more precocious in the group with moderate-to-severe symptoms at baseline. One possible reason is that the initiation of treatment with LAI in the group with the greatest severity may have led to a quicker stabilization of symptomatology and acquisition of illness insight, and thus to a better adherence to oral medication and to a more positive attitude toward treatment. On the contrary, the group already in remission or with mild symptoms (BPRS <41 at baseline) may have had only subtle changes in the first period of treatment with better improvements in the long-term. This is also confirmed by the LOCF analyses that showed no significant associations between predictors and symptoms changes over time in the group with BPRS <41 at baseline.

Overall, our findings lead to a critical reflection on the importance of the quality of relationship between clinicians and patients and the necessity to provide information regarding the potential benefits of psychotropic medications, including LAIs. Unfortunately, real-world psychiatric services often lack sufficient resources—such as availability of time and personnel—to adequately inform and support the needs of people suffering from severe mental illnesses. For instance, a study by Potkin et al. (58) reported that clinicians spent an average time of 12 min with patients in treatment with LAIs, and that only 2% of this time was occupied by discussions about the adherence to medication. However, shared-decision making has been extensively suggested as a winning strategy to overcome barriers hampering the acceptance of LAIs by patients and improve the outcome (37).

The main limitation of the present study is that data were drawn from a naturalistic, observational study about the use of LAIs in real-word clinical practice. Therefore, our results cannot be generalized to patients taking oral medications only, a situation in which patients' attitude and adherence to medication may play a more relevant role in symptoms changes. Moreover, our analyses did not take into account some important factors, such as the type of psychiatric diagnosis, the type of oral medication taken by participants, the duration of untreated illness, and the number of previous oral medication. However, a stratification according to these factors may have substantially reduced the sample size and thus the validity of our findings. Besides attitude toward treatment and adherence, future research should adopt tools evaluating the quality of patient-clinician relationship as well as insight levels to investigate more in-depth the complex associations between these factors and clinical response in patients with severe mental illnesses.

In conclusion, the present study did not confirm our initial hypothesis that attitude toward medication and treatment adherence may represent potential predictors of changes in psychiatric symptoms over time in a naturalistic sample. However, severity represents a key factor, as patients with the most severe symptoms may benefit more precociously from LAI treatment due to improvement in attitude toward medication and treatment adherence. Our findings remark that it is fundamental to build and maintain a meaningful and reciprocal therapeutic alliance with patients suffering from severe mental illnesses over time. Such a relationship should involve a clear and comprehensive explanation of both the efficacy and the potential side effects of psychotropic drugs, including LAI, to convey greater awareness to the patients (38). A focus on patients' experience and preferences could help improve attitudes toward and appropriate use of LAI antipsychotics (29, 59). The increase of resources allocated to mental health services should be considered a priority to improve the long-term outcomes for psychiatric patients thus reducing the economic burden of chronic mental illnesses.

## Data Availability Statement

The raw data supporting the conclusions of this article will be made available by the authors, without undue reservation.

## Ethics Statement

The study protocol was approved by the local ethics committee of the coordinating centre (Ethics Committee for Clinical Trials of the Provinces of Verona and Rovigo, protocol n.57622 of the 09/12/2015) and of each participating centre. The participants provided their written informed consent to participate in this study.

## Author Contributions

AAg and LF-P: statistical analyses and writing original draft. VP, AAm, and CC: data collection and revision of data literature. GM, GC, FB, and AD'A: supervision data collection, review and editing of the original draft, and scientific advisor of the project. GS, EA, and MA: review and editing of the original draft. CB and GO: writing protocol, designed the study, and scientific advisor of the project. All authors approved of the final draft of the manuscript before submission.

## Funding

This work was supported by the Youth Foundation, Hong Kong (YF2012B-HX01).

## Conflict of Interest

GM has been a consultant and/or a speaker and/or has received research grants from Angelini, Doc Generici, Janssen, Lundbeck, Otsuka, and Pfizer. The remaining authors declare that the research was conducted in the absence of any commercial or financial relationships that could be construed as a potential conflict of interest.

## Publisher's Note

All claims expressed in this article are solely those of the authors and do not necessarily represent those of their affiliated organizations, or those of the publisher, the editors and the reviewers. Any product that may be evaluated in this article, or claim that may be made by its manufacturer, is not guaranteed or endorsed by the publisher.
